# Supplementary material for: Mars’ induced magnetosphere can form under radial interplanetary magnetic field
Source: Innovation (Camb). 2026 Feb 11;7(6):101312. doi: 10.1016/j.xinn.2026.101312 (PMC13237844; doi:10.1016/j.xinn.2026.101312)
Supplement: Document S1. Figures S1–S6 [file mmc1.pdf]

The Innovation, Volume 7

Supplemental Information

Mars' induced magnetosphere can form under radial interplanetary magnetic field

Rentong Lin, Shiyong Huang, Jingyi Zhou, Yuming Wang, Zhigang Yuan, Eduard Dubinin, Markus Fränz, Haoyu Lu, Kaijun Liu, Lihui Chai, Yihui Song, Guoqiang Wang, Yutian Chi, Honghong Wu, Kui Jiang, Qiyang Xiong, and Zhuxuan Zou

Supplementary Information

Supplemental Figures

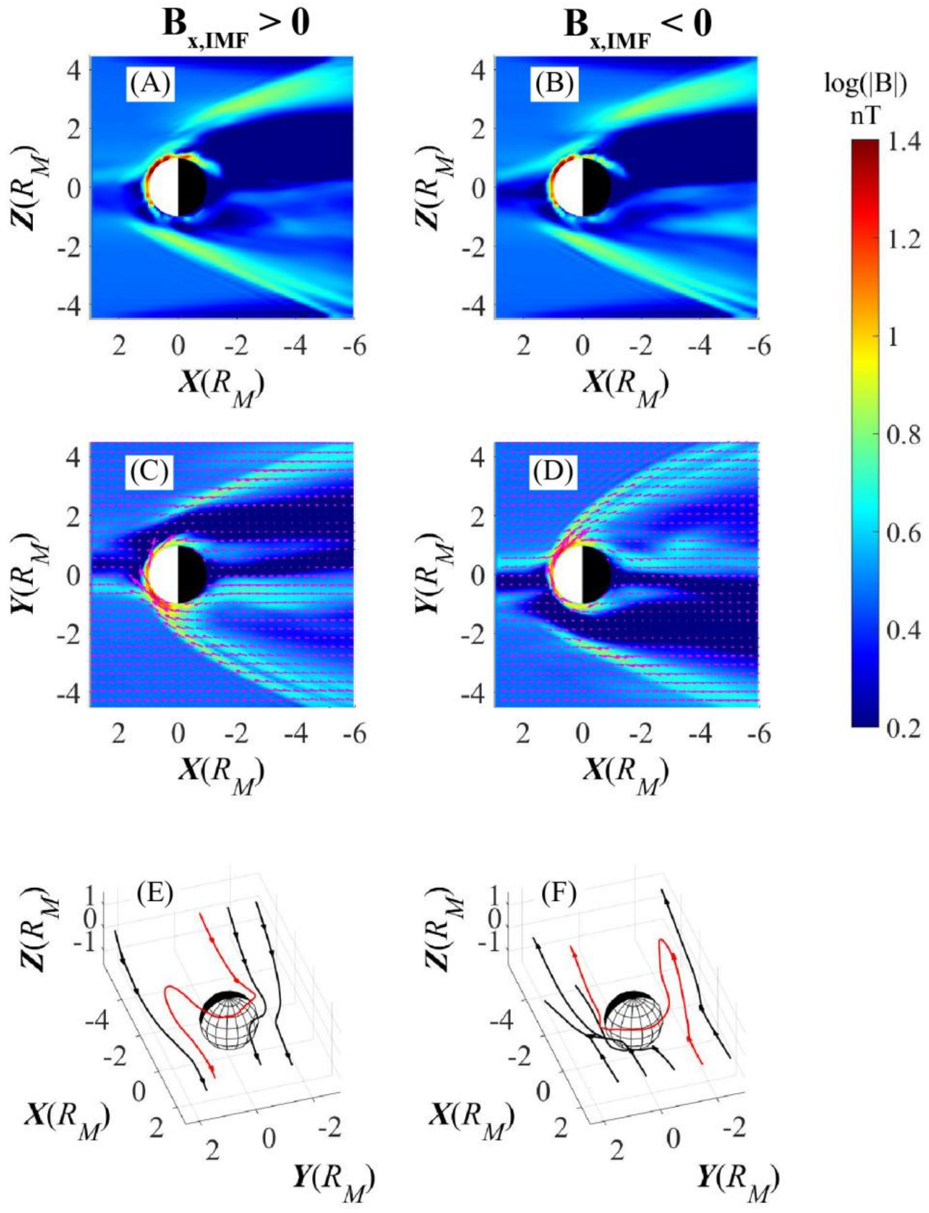

Figure S1. Magnitude of magnetic field in X-Z plane and X-Y plane in MSE coordinate and magnetic field lines from hybrid simulations. Panel a presents distribution of magnetic field magnitude when $B_{x,IMF} > 0$ and Panel b presents that when $B_{x,IMF} < 0$ in X-Z plane (Y = 0). Panel c presents distribution of magnetic field magnitude when $B_{x,IMF} > 0$ and Panel d presents that when $B_{x,IMF} < 0$ in X-Y plane (Z = 0). Panel e presents distribution of magnetic field lines when $B_{x,IMF} > 0$. The magnetic field lines originate from a group of points with the X = -6 R_M and Z = 0 R_M but different Y values. And Panel f presents distribution of magnetic field lines when $B_{x,IMF} < 0$. The magnetic field lines originate from a group of points with the X = 3 R_M and Z = 0 R_M but different

Y values. The red streamlines in panel e and f represent draped magnetic fields, corresponding to the blue curves in Fig 5.

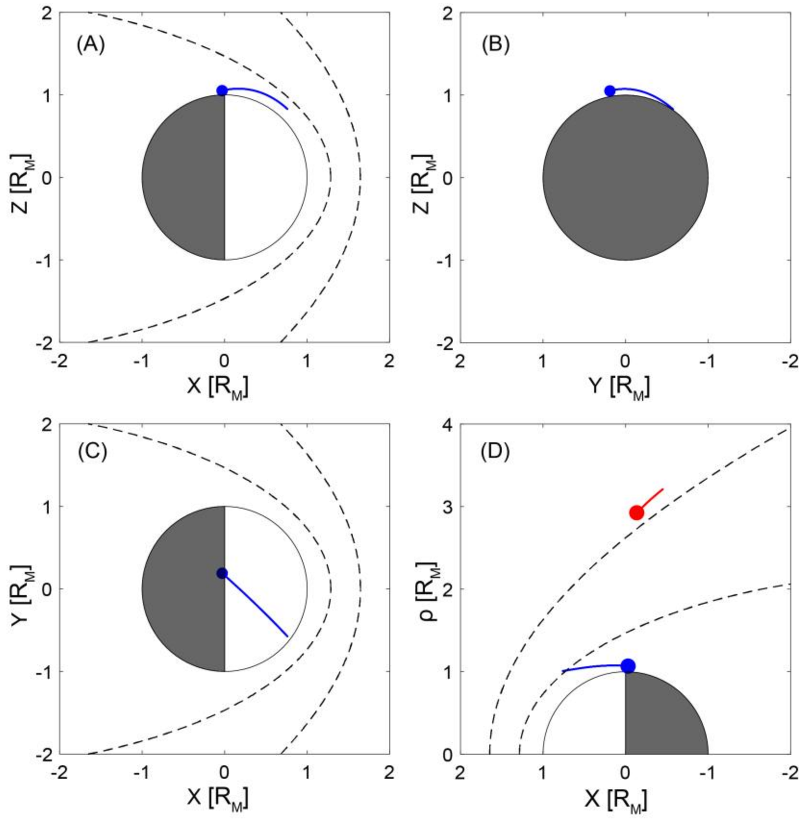

Figure S2. Orbits of Tianwen-1 and MAVEN orbiters in the time interval of 09:23-09:40 UT on June 5, 2023. Orbits of MAVEN (a) in X-Z plane from the dawn, (b) in Y-Z plane viewed from the nightside, (c) in X-Y plane from the north pole, and (d) in X-ρ plane, where $\rho = \sqrt{y^2 + z^2}$. The blue curve represents orbit of MAVEN and the red one represents orbit of Tianwen-1. The blue and red dots represent start of the orbit of orbiters, respectively. The two dashed curves represent the bow shock and the induced magnetosphere boundary from the model (Vignes et al., 2000), respectively. The circle in (a-c) and the half of black circle in (d) represents Mars.

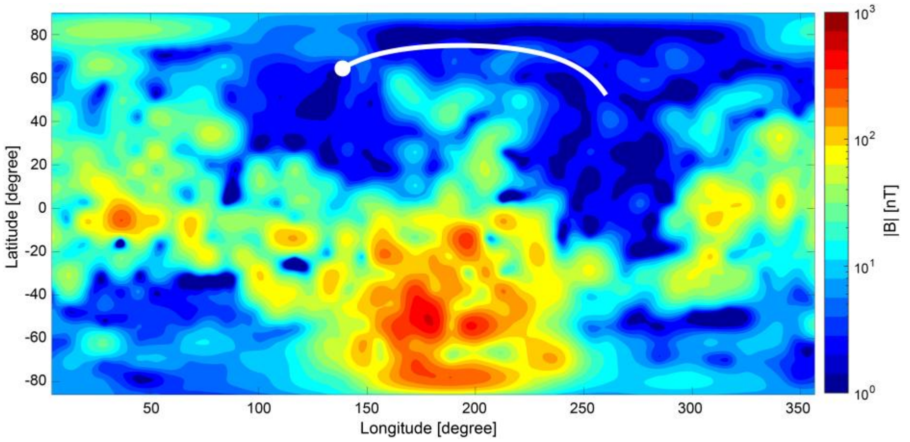

Figure S3. The contour map represents the magnitude of crustal magnetic field at altitude of 185 km by model (Morschhauser et al., 2014). The white dot represents the start of the orbit of MAVEN at 09:15 UT and the white curve represents the orbit of MAVEN in the time interval of 09:15-09:48 UT on June 5, 2023.

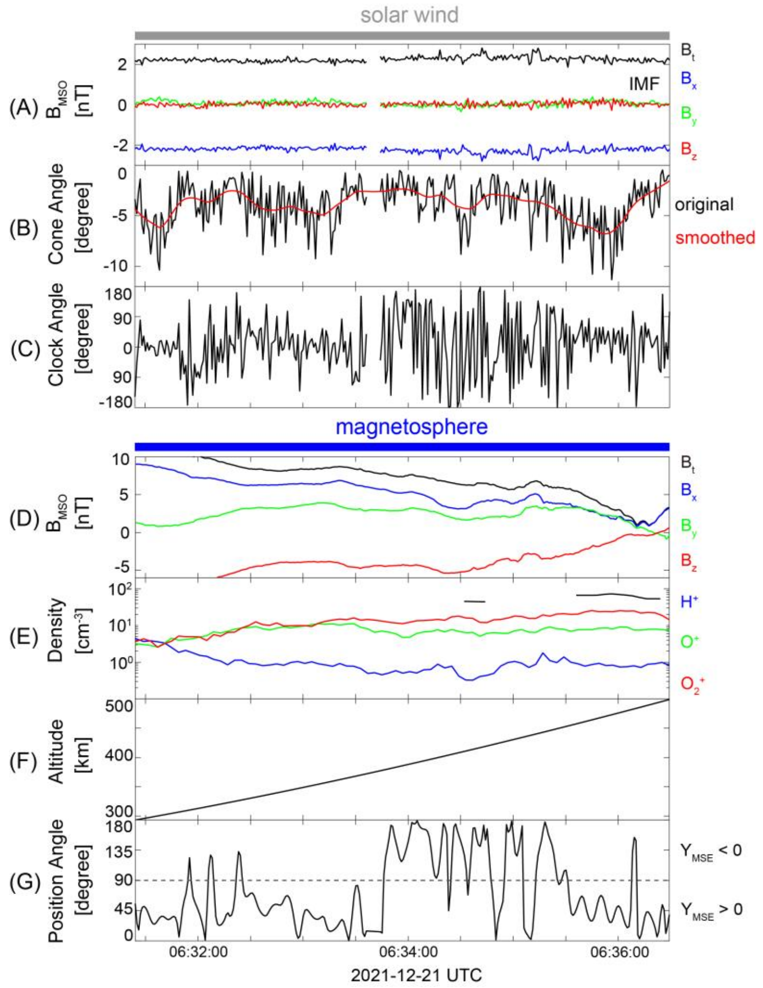

Figure S4. Magnetic field and plasmas measurements by MAVEN, accompanying by

the interplanetary magnetic field (IMF) measurements by Tianwen-1 in the time interval of 06:31:25-06:36:30 UT on December 21, 2021. (a) IMF in MSO coordinate by Tianwen-1, (b-c) cone angle and clock angle of IMF, (d) magnetic field in MSO coordinate by MAVEN, (e) ion density, (f) altitude, and (g) position angle between position vector of MAVEN and IMF projection vector in the Y-Z plane. The red curve in panel (b) represents the cone angle of IMF smoothed over 30 data points with 1Hz sampling rate of magnetic field. Position angle is defined as $\cos^{-1}\left(\frac{\boldsymbol{P}_{yz,MAVEN}\cdot\boldsymbol{B}_{yz,Tianwen-1}}{|\boldsymbol{P}_{yz,MAVEN}||\boldsymbol{B}_{yz,Tianwen-1}|}\right)$, where $\boldsymbol{P}_{\mathrm{yz,MAVEN}} = [\mathrm{Y}, \mathrm{Z}]\ \mathrm{R_M}$ and $\boldsymbol{B}_{\mathrm{yz,Tianwen\text{-}1}} = [B_{\mathrm{y}}, B_{\mathrm{z}}]$ nT in MSO coordinate. Note that the angle lower than 90 degrees (higher than 90 degrees) represents that MAVEN is in hemisphere where Y > 0 (Y < 0) in MSE coordinate.

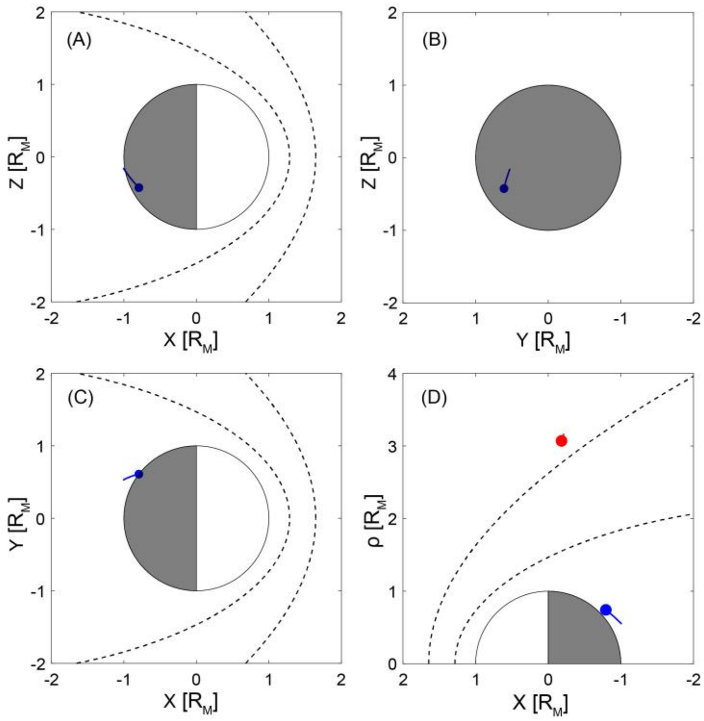

Figure S5. Orbits of Tianwen-1 and MAVEN orbiters in the time interval of 06:31:25-06:36:30 UT on December 21, 2021. Orbits of MAVEN (a) in X-Z plane from the dawn, (b) in Y-Z plane viewed from the nightside, (c) in X-Y plane from the north pole, and (d) in X-ρ plane, where $\rho = \sqrt{y^2 + z^2}$. The blue curve represents orbit of MAVEN and the red one represents orbit of Tianwen-1. The blue and red dots represent start of the orbit of orbiters, respectively. The two dashed curves represent the bow shock and the induced magnetosphere boundary from the model (Vignes et al., 2000), respectively. The circle in (a-c) and the half of black circle in (d) represents Mars.

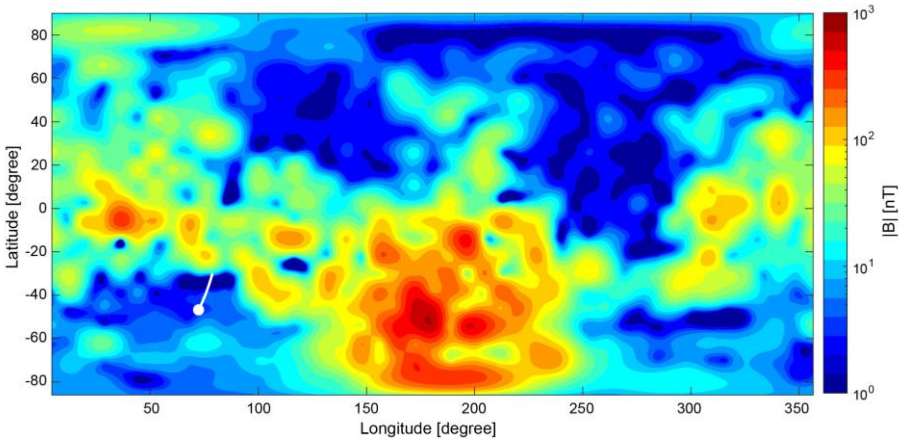

Figure S6. The contour map represents the magnitude of crustal magnetic field at altitude of 185 km by model (Morschhauser et al., 2014). The white dot represents the start of the orbit of MAVEN at 06:31:25 UT and the white curve represents the orbit of MAVEN in the time interval of 06:31:25-06:36:30 UT on December 21, 2021.
