# Supplementary material for: Mars’ induced magnetosphere can form under radial interplanetary magnetic field
Source: Innovation (Camb). 2026 Feb 11;7(6):101312. doi: 10.1016/j.xinn.2026.101312 (PMC13237844; doi:10.1016/j.xinn.2026.101312)
Supplement: Document S2. Article plus supplemental information [file mmc2.pdf]

REPORT | Planetary Sciences

OPEN ACCESS

www.the-innovation.org

Mars' induced magnetosphere can form under radial interplanetary magnetic field

Rentong Lin,[1,3,4] Shiyong Huang,[1,*] Jingyi Zhou,[2,3,4] Yuming Wang,[3,4] Zhigang Yuan,[1] Eduard Dubinin,[5] Markus Fränz,[5] Haoyu Lu,[6] Kaijun Liu,[2] Lihui Chai,[7,8] Yihui Song,[6] Guoqiang Wang,[9] Yutian Chi,[3] Honghong Wu,[1] Kui Jiang,[1] Qiyang Xiong,[1] and Zhuxuan Zou[3,4]

*Correspondence: shiyonghuang@whu.edu.cn

Received: September 25, 2025; Accepted: February 6, 2026; Published Online: February 11, 2026; https://doi.org/10.1016/j.xinn.2026.101312

© 2026 The Author(s). Published by Elsevier Inc. on behalf of Youth Innovation Co., Ltd. This is an open access article under the CC BY license (http://creativecommons.org/licenses/by/4.0/).

GRAPHICAL ABSTRACT

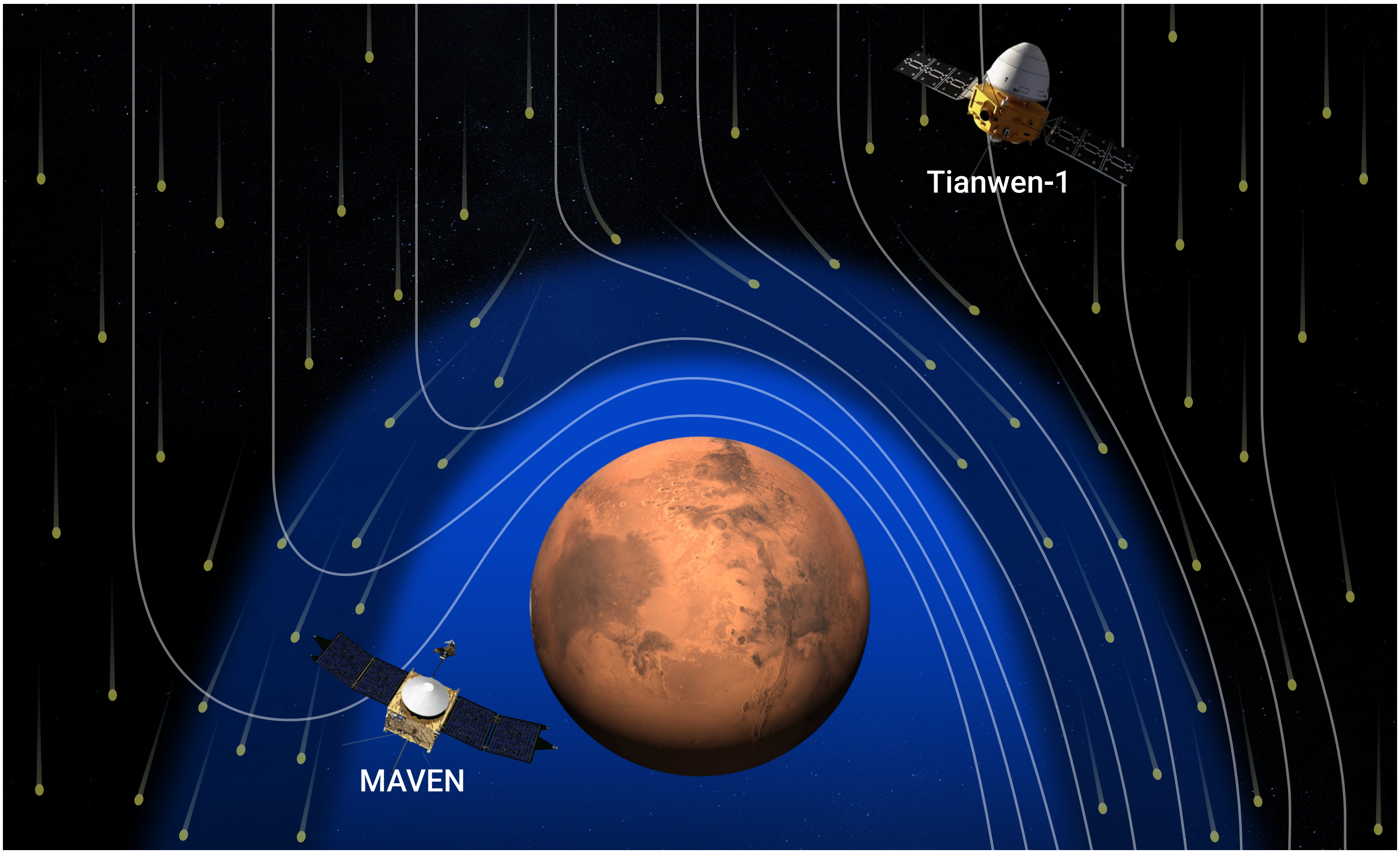

PUBLIC SUMMARY

- Magnetic barrier above Mars' ionosphere is observed and simulated under radial interplanetary magnetic field.
- A conductive ionosphere ensures interplanetary magnetic field's draping around Mars.
- This pattern can be referred to exoplanets near the dwarf stars.

Mars' induced magnetosphere can form under radial interplanetary magnetic field

Rentong Lin,[1,3,4] Shiyong Huang,[1,*] Jingyi Zhou,[2,3,4] Yuming Wang,[3,4] Zhigang Yuan,[1] Eduard Dubinin,[5] Markus Fränz,[5] Haoyu Lu,[6] Kaijun Liu,[2] Lihui Chai,[7,8] Yihui Song,[6] Guoqiang Wang,[9] Yutian Chi,[3] Honghong Wu,[1] Kui Jiang,[1] Qiyang Xiong,[1] and Zhuxuan Zou[3,4]

[1]School of Earth and Space Science and Technology, Wuhan University, Wuhan 430072, China
[2]Department of Earth and Space Sciences, Southern University of Science and Technology, Shenzhen 518055, China
[3]National Key Laboratory of Deep Space Exploration/School of Earth and Space Sciences, University of Science and Technology of China, Hefei 230026, China
[4]CAS Center for Excellence in Comparative Planetology/CAS Key Laboratory of Geospace Environment/Mengcheng National Geophysical Observatory, University of Science and Technology of China, Hefei 230026, China
[5]Max-Planck-Institute for Solar System Research, 37077 Göttingen, Germany
[6]School of Space and Earth Sciences, Beihang University, Beijing 102206, China
[7]Key Laboratory of Earth and Planetary Physics, Institute of Geology and Geophysics, Chinese Academy of Sciences, Beijing 100029, China
[8]College of Earth and Planetary Sciences, University of Chinese Academy of Sciences, Beijing 100049, China
[9]Institute of Space Science and Applied Technology, Harbin Institute of Technology, Shenzhen 518000, China
*Correspondence: shiyonghuang@whu.edu.cn

Received: September 25, 2025; Accepted: February 6, 2026; Published Online: February 11, 2026; https://doi.org/10.1016/j.xinn.2026.101312

© 2026 The Author(s). Published by Elsevier Inc. on behalf of Youth Innovation Co., Ltd. This is an open access article under the CC BY license (http://creativecommons.org/licenses/by/4.0/).

Citation: Lin R., Huang S., Zhou J., et al., (2026). Mars' induced magnetosphere can form under radial interplanetary magnetic field. The Innovation **7(6)**, 101312.

The interaction between planetary atmosphere and stellar winds governs atmospheric evolution in unmagnetized planets. Generally, an interplanetary magnetic field (IMF) drapes around the planetary ionosphere, creating a magnetic barrier that deflects stellar winds and leads to the formation of an induced magnetosphere. However, whether an induced magnetosphere can form under radial IMF conditions where the IMF aligns with solar wind flow in our Solar System remains controversial. By analyzing joint observations from the Tianwen-1 orbiter and the Mars Atmosphere and Volatile Evolution mission combined with hybrid numerical simulations, we clearly demonstrate the formation of Mars' induced magnetosphere during the radial IMF for the first time. This induced magnetosphere comprises a draped magnetic field and an induced magnetic field. Magnetic pressure buildup above the ionosphere surpasses incident solar wind pressure, which establishes a stable magnetic barrier. This finding indicates that the draped magnetic field still forms under a radial IMF. The formation of Mars' induced magnetosphere under the radial IMF could be a general pattern for the interaction between the IMF and planetary atmosphere, which can be referred to terrestrial exoplanets within the close-in habitable zone of dwarf stars. This work clarifies the fundamental understanding of solar wind interactions with unmagnetized planets across diverse solar wind conditions.

INTRODUCTION

Previous Mars exploration missions have shown that Mars possesses an atmosphere but lacks a global intrinsic magnetic field.[1] The atmosphere of Mars is partially ionized by solar radiation, leading to the formation of an ionosphere. This ionosphere blocks and deflects the solar wind, within which the interplanetary magnetic field (IMF) is embedded. The IMF drapes around the upper boundary between the ionosphere and the solar wind, forming a draped magnetic field. Induced currents can be generated in the boundary and within the ionosphere, which produces an induced magnetic field. The draped field and the induced field constitute the magnetic field in Mars' induced magnetosphere.[2,3] The draped magnetic field arises from mass loading of the solar wind, and equals the induced magnetic field generated by the motional electric field in the interaction of the conductive ionosphere and upstream plasmas. The induced magnetic field results from the diamagnetic field generated by the diamagnetic current in the ionosphere, which counteracts the penetration of the external IMF.

The draped magnetic field[4–14] and the induced magnetic field[3,15] have been widely identified on unmagnetized planets in previous observations and simulations. However, their magnitudes strongly depend on solar wind conditions. It has been suggested that the draped field component is expected to be small when the IMF cone angle, i.e., the angle between the solar wind velocity and the IMF, is small due to the weak motional electric field.[16] In this case, if the induced field's magnitude is also small, i.e., lower than the pristine IMF, the magnetic barrier would be absent and the induced magnetosphere degenerates; conversely, if either the draped field or the induced field is present (magnitude larger than the IMF), there should be a magnetic barrier between the ionosphere and the shocked solar wind and the characteristics of the draped field or induced field can be detected.

Numerous studies have been conducted on this topic. DeZeeuw et al.[17] proposed a two-dimensional magnetohydrodynamic (MHD) model, suggesting that Venus' magnetotail is not induced under radial IMF conditions. Zhang et al.[18] performed a case study and an MHD simulation with an IMF cone angle of 11°, observing a notable absence of the dayside portion of Venus' induced magnetosphere. Similarly, Zhang et al.[16] employed hybrid simulations with an IMF cone angle of 4° and proposed that Mars' induced magnetosphere degenerates. However, using magnetic field and plasma measurements from Venus Express, Dubinin et al.[3] identified induced magnetic fields and a circular plasma sheet on Venus under radial IMF conditions. Rong et al.[19] found that Venus still has an evident induced magnetosphere even if the IMF is nearly radial. Fowler et al.[20] studied Mars' deep ionosphere under radial IMF conditions and determined that magnetic pressure can counterbalance the solar wind dynamic pressure.

Therefore, the physical picture remains controversial and the existence of induced currents and corresponding magnetic fields, as well as the potential degeneration of Mars' induced magnetosphere under a radial IMF, remains unconfirmed and unquantified by observations. Given that the response time of Mars' magnetosphere to the IMF orientation is less than 10 min,[21,22] simultaneous observations of the solar wind and the magnetosphere are necessary to identify the induced magnetosphere under radial IMF conditions.

Although there have been periods when multiple satellites orbited Mars, no two magnetometer-equipped orbiters have been operated simultaneously. This limitation prevented the simultaneous observation of the IMF and the magnetic field of the induced magnetosphere. Joint observations from China's Tianwen-1 mission,[23–25] which entered the orbit of Mars in 2021, and the US Mars Atmosphere and Volatile Evolution (MAVEN) satellite,[26] which has been in service since 2014, now enable simultaneous observations of the upstream solar wind and the downstream region, including Mars' magnetosheath and induced magnetosphere.[27] Using joint observations from the Tianwen-1 in the solar wind and MAVEN in the induced magnetosphere, we clearly identified a magnetic barrier between Mars' ionosphere and shocked solar wind under the radial IMF for the first time. Draped magnetic fields near Mars are demonstrated in a case study and statistical analysis.

MATERIALS AND METHODS

Instruments

The data used in this study are from the Tianwen-1 and MAVEN missions, particularly from the Mars Orbiter Magnetometer (MOMAG)[25,28] onboard Tianwen-1, the Magnetometer (MAG),[29] the Solar Wind Electron Analyzer (SWEA),[30] and the Supra-Thermal And Thermal Ion Composition (STATIC)[31] onboard MAVEN. The 2C-level data of MOMAG are used in this study. MOMAG and MAG provide 3D magnetic fields with a cadence of 32 vectors s^{-1}.

The reliability of MOMAG has been verified by Zou et al.[32] and Wang et al.[33] The absolute vector accuracy of data from MAG is 0.05%.[29] SWEA provides an electron energy spectrogram and pitch angle distribution at a temporal resolution of 2 s. STATIC provides an energy spectrogram of ions at a temporal resolution of 4 s and ion velocity at a time resolution of 16 s. Note that the ion density and velocity vectors are derived from STATIC's measurements.

The Tianwen-1 spacecraft is in an elliptical orbit with a periapsis altitude of ~270 km and apoapsis altitude of ~11,000 km with a period of ~8 h, and the MAVEN spacecraft is orbiting Mars with a periapsis altitude of ~170 km and apoapsis altitude of ~4,400 km with a period of ~4.5 h from November 13, 2021, to October 31, 2023. It is advantageous for Tianwen-1 to take long-time measurements of the solar wind.

Coordinates

In Mars-solar-orbital (MSO) coordinates, the X axis points from Mars toward the Sun, the Y axis points opposite to the direction of the orbital velocity component of Mars perpendicular to the X axis, and the Z axis completes the orthogonal coordinate set.

In Mars-solar-electrical (MSE) coordinates, the X axis points to the Sun from Mars (assuming that the velocity of solar wind $\boldsymbol{V}_{SW}$ points from the Sun to Mars), and the Z axis points to the convective electric field $\boldsymbol{E}$, where $\boldsymbol{E} = -\boldsymbol{V}_{SW} \times \boldsymbol{B}_{IMF}$, $\boldsymbol{B}_{IMF}$ is the IMF, and the Y axis completes the right-hand system.

Pressure terms obtained from MAVEN measurements

Four different pressure terms are considered in this study and are estimated using the following equations.[34] The magnetic pressure $P_{magnetic,MAG}$ is obtained from magnetic field $\boldsymbol{B}$ from MAG via $P_{magnetic,MAG} = \frac{\boldsymbol{B}^2}{2\mu_0}$, where μ_0 is the vacuum permeability. The thermal pressure $P_{thermal,LPW}$ is estimated from the electron density N_e and temperature T_e from the Langmuir Probe and Waves (LPW) instrument, assuming $N_e \approx N_i$ and $T_e \approx T_i$ because the temperature of heavy ions, especially for low-energy ions in Mars' ionosphere, is difficult to measure.[31] $P_{thermal,LPW}$ is obtained using $P_{thermal,LPW} = 2N_e k_B T_e$, where k_B is the Boltzmann constant. The thermal pressure in the magnetosheath, $P_{thermal,\ SWIA}$, is estimated from the ion density N_i and temperature T_i from SWIA, assuming $T_e \ll T_i$. $P_{thermal,\ SWIA}$ is obtained using $P_{thermal,\ SWIA} = N_i k_B T_i$. The solar wind dynamic pressure $P_{dynamic,\ SWIA}$ is estimated from the ion density N_i and velocity $\boldsymbol{V}_i$ from SWIA using $P_{dynamic,SWIA} = m_p N_i \boldsymbol{V}_i^2$, where m_p is the proton mass. It should be noted that the thermal pressure $P_{thermal,LPW}$ estimated by LPW can only apply in the induced magnetosphere and ionosphere, and $P_{thermal,\ SWIA}$ by SWIA in the solar wind and the magnetosheath. Notably, to minimize the impact of crustal magnetic fields, measurements in strong crustal field regions are excluded. Strong crustal field regions are defined as regions where the magnitude of the crustal magnetic field greater than 10 nT at an altitude of 185 km according to a previous model.[35]

Introduction of the hybrid simulation model

The three-dimensional simulations in this study were conducted using the RHybrid simulation platform, as described in Jarvinen et al.[36] and Zhou et al.[37] This code employs a macroparticle description for ions and a massless, charge-neutralizing fluid model for electrons. The simulations use the MSO coordinate system, neglecting the minor aberration caused by Mars's motion around the Sun. Two simulations were performed under two $B_{x,\ radial\ IMF}$ conditions, with the same simulation domain: $-6\ R_M < X < 3\ R_M$, $-6\ R_M < Y < 6\ R_M$, and $-4\ R_M < Z < 4\ R_M$. The simulation domain is partitioned into uniform cubic cells with a side length of 0.08 R_M, and the simulation timestep is 20 ms. The upstream solar wind has a number density of 4 cm^{-3}, a temperature of 1×10^5 K, and a -X-directional bulk velocity of 380 km/s. The number of macroparticles per simulation grid is 500. The upstream IMF is set at a fixed magnitude of 3 nT and with the vector lying in the X-Y plane under two conditions: (2.993, 0.21, 0) nT and (−2.993, 0.21, 0) nT, corresponding to cone angles of 4° and −4°, respectively.

The Mars model consists of a sphere centered at the origin with a radius of 3,390 km, representing the solid planet. An obstacle located 300 km above the Martian surface is modeled as a superconducting sphere, representing the ionospheric barrier to solar wind flow. The resistivity, electron velocity, and pressure within this obstacle are set to zero, whereas outside the obstacle, a resistivity of 5,000 Ω·m is applied to add magnetic field diffusion to improve numerical stability. For simulation runs in which the IMF flow-aligned component is nonzero, a scalar potential field corresponding to the magnetic field around an ideally conducting sphere is used to implement the flow-aligned IMF component. This constant field is zero inside the obstacle.

The crustal magnetic fields are not included in the simulations. The planetary ions consist of O^+, and O_2^+ from the ionosphere and H^+ and O^+ from the exosphere. The ionospheric planetary ions are emitted from a spherical inner boundary 400 km above the surface of Mars, with a cosine-dependent emission rate on the solar zenith angle on the dayside and a constant value at night. It peaks at noon and gradually decreases to just 10% of its noon-time peak intensity at the terminator. The total ionospheric emission rates were chosen to best fit the spacecraft observations analyzed here. On the other hand, exospheric planetary ions are produced through photoionization, which is based on the neutral profiles of "Run B (solar min, with exosphere)" from the ISSI team's second meeting.[36] For simplicity, the photoionization rate is set as a constant above the inner boundary but is absent within Mars's shadow. The emission rates for all ions follow the setups described in Zhou et al.[37] Additionally, there is a spherical, absorbing inner boundary 200 km above the Mars surface. The particles that reach this boundary are removed from the system. All simulation parameters are stationary in the runs analyzed here.

Criterion of radial IMFs and valid observations

The cone angle of the IMF is defined as $\theta = \operatorname{atan}\left(\frac{\sqrt{B_y^2+B_z^2}}{B_x}\right)$, where B_x, B_y, B_z are IMF components in MSO coordinates. A positive cone angle ($\theta > 0$) means that the B_x component of the IMF is positive ($B_x > 0$) or vice versa. When the cone angle of the IMF remains in the range of −15° to 0 or 0° to 15° for 240 s, the IMF is approximately radial. Magnetic field and plasma measurements by MAVEN at the midpoint of these 240 s are regarded as valid observations in the case of radial IMFs and are included in the statistical analysis. If the IMF observed by Tianwen-1 remains quasi-radial within 240 s, the magnetospheric magnetic field observed by MAVEN downstream is considered to have recovered to the condition under the quasi-radial IMF. One should note that we have to apply less-strict constraints on the IMF cone angle to obtain statistical results since there are an insufficient number of events with cone angles less than 5°.

Note that the cone angle should also depend on the direction of the solar wind velocity. Unfortunately, owing to limitations in the field of view of MINPA and the orbit of Tianwen-1, calculating the solar wind velocity vector using MINPA data remains challenging. The orbital motion of Mars results in an ~4° deviation from the radial direction at normal solar wind velocity. Most cases are not excluded since the deviation (~4°) is much smaller than the upper limit of this criterion (±15°). If the solar wind velocity changes or has a significant nonradial component, some cases based on this criterion should be excluded. However, significant changes in the direction of the solar wind velocity are rare in the orbit of Mars. In addition, cases of changes in solar wind are accompanied mostly by disturbances in the IMF, which are excluded from the dataset used in this study. Thus, the assumption that the direction of the solar wind is along the X axis should not impact the results or the main conclusions.

Estimating current density using the Maxwell-Ampere law

As described by the Maxwell-Ampere law, the curl of the local magnetic field, $\boldsymbol{B}$, at any point in space is shaped by the local current density $\boldsymbol{J}$, i.e., $\nabla \times \boldsymbol{B} = \mu_0 \boldsymbol{J} + \varepsilon_0 \mu_0 \frac{\partial \boldsymbol{E}}{\partial t}$, where $\varepsilon_0 \mu_0 \frac{\partial \boldsymbol{E}}{\partial t}$ is the displacement current and where ϵ_0 and μ_0 are the electromagnetic permittivity and permeability in a vacuum, respectively. Considering the nonrelativistic regime and assuming electric charge quasi-neutrality, which is well justified at large scales of interest, the displacement current contribution can be neglected.[38] Therefore, the local current density given by $\boldsymbol{J} = \frac{1}{\mu_0} \nabla \times \boldsymbol{B} \approx \frac{\Delta \boldsymbol{B}}{\mu_0 \Delta \boldsymbol{r}}$, where $\Delta \boldsymbol{B}$ represents the difference in the magnetic field and where Δr represents the space scale, providing a "zeroth-order" scale estimation.

Estimating current density derived from momentum equations

In the upper ionosphere of Mars, electrical currents can be derived from the momentum equations for the ions and electrons. The current density $\boldsymbol{J}$ perpendicular to magnetic field $\boldsymbol{B}$ can be described as $\boldsymbol{J}_\perp = \frac{\boldsymbol{B}\times}{B^2}\left(\nabla P + \frac{d(\rho \boldsymbol{V})}{dt}\right)$, neglecting the collisional term, where P is the plasma pressure, ρ is the mass density, and $\boldsymbol{V}$ is the plasma bulk velocity.[3] For quasistatic equilibrium, the expression is reduced to $\boldsymbol{J}_\perp = \frac{\boldsymbol{B}\times\nabla P}{B^2} \approx \frac{\Delta P}{|\boldsymbol{B}|\Delta r}$, where ΔP is the change in plasma pressure, $\boldsymbol{B}$ is the magnetic field, and Δr represents the space scale. To estimate the current at the magnetic pile-up boundary (MPB), estimation of thickness of the MPB is needed. To calculate the thickness of the MPB, one should identify the upper boundary and the lower boundary of the MPB. The upper boundary is the transition point from a thermal pressure-dominated regime in the magnetosheath to a magnetic pressure-dominated one. The lower boundary is where the magnetic field intensity reaches its maximum value. The thickness of the MPB can be estimated by the difference of altitude of the upper boundary and the lower boundary; the estimated current density derived from this method is essentially a diamagnetic current density.

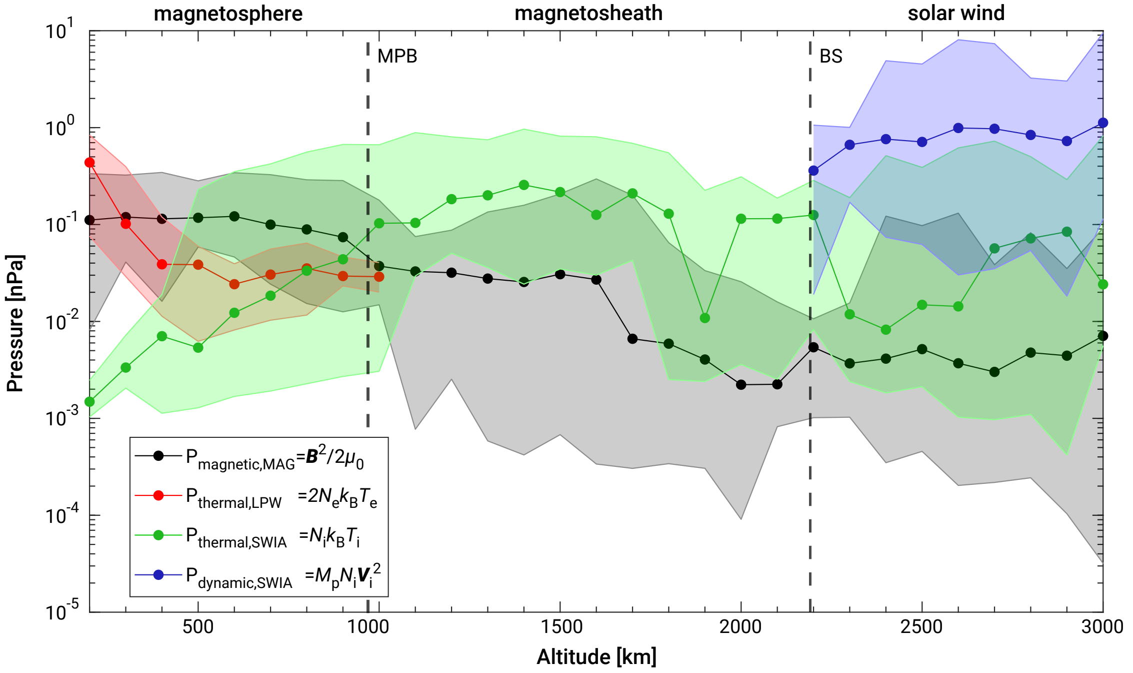

Figure 1. Different pressure terms on the dayside corresponding to altitude in the case of radial IMFs The black dots represent median value of magnetic pressure ($P_{magnetic,MAG}$). The red dots represent median value of thermal pressure estimated by LPW data ($P_{thermal,LPW}$). The green dots represent median values of thermal pressure estimated by SWIA data ($P_{thermal,\ SWIA}$). The blue dots represent median values of solar wind dynamic pressure ($P_{dynamic,\ SWIA}$). The shaded areas represent the range between the maximum and minimum measurement values. The vertical dashed lines mark the MPB and the bow shock (BS) stand-off distances at 1.29 and 1.64 R_M (radius of Mars).[39] Note that here the median values were used instead of mean values because of the advantage of the median over the mean for its greater resistance to outliers and stronger stability. The median is more suitable for describing the central tendency of data with outliers or skewed distributions.

RESULTS

We surveyed for simultaneous observations under radial IMFs with cone angles between −15° and 15° from November 13, 2021, to October 31, 2023. We observed 79 cases of radial IMFs with 38,387 magnetic field datapoints at a sampling rate of 1 Hz. Note that the effect of the crustal field is excluded in this study (see more details in materials and methods). The detailed criterion of the radial IMFs and valid observations are described in the materials and methods.

Figure 1 presents different pressure terms on the dayside from MAVEN measurements in the case of radial IMFs. Details of the estimation of each pressure term can be found in the materials and methods. The solar wind dynamic pressure $P_{dynamic,\ SWIA}$ and thermal pressure $P_{thermal,\ SWIA}$ dominate the solar wind and the magnetosheath, and sharply decrease near the MPB. The magnetic pressure $P_{magnetic,MAG}$ becomes the main pressure term in regions between the MPB and the deep ionosphere (∼200 km). The thermal pressure $P_{thermal,LPW}$ dominates in the deep ionosphere. This finding suggests that a magnetic barrier exists between the shocked solar wind and Mars' ionosphere.

Another criterion for identifying the existence of an induced magnetosphere is whether the IMF is draped around the planet and forms a draping magnetic field. The direction of the draping magnetic field depends on the component of the IMF perpendicular to the solar wind velocity. In the MSE coordinates, this perpendicular component always aligns with the +Y axis. Therefore, if there exists an induced magnetosphere, the draping magnetic field consistently points toward the magnetotail ($B_x < 0$) in the hemisphere where Y > 0, and toward the Sun ($B_x > 0$) in the hemisphere where Y < 0 in the MSE coordinates. Otherwise, the direction of magnetic field near the planet, except for the crustal magnetic fields, should all align with that of the IMF.

Table 1 displays the B_x component of the magnetic field in different regions with different radial IMFs. When the B_x component of the IMF is positive, as observed by Tianwen-1, the magnetic field B_x component, measured by MAVEN in the solar wind, is predominantly positive as well, and vice versa. Within the magnetosheath, a small fraction of the B_x component opposes the direction of the IMF. Below the MPB, nearly half or even more of the B_x component is oriented opposite to the IMF. These findings indicate the presence of plasma currents and global-scale magnetic field structures in the region near and below the MPB.

Figure 2 displays the averaged magnetic field B_x component from observations in MSE coordinates and hybrid simulations without crustal magnetic fields. At higher altitudes (altitude ≥ 0.5 R_M), the magnetic field generally aligns with the direction of the IMF. However, at lower altitudes (altitudes < 0.5 R_M), the B_x component of the magnetic field intensifies and the direction of the magnetic field varies in different hemispheres: in the hemisphere where Y > 0, the B_x component is predominantly negative, whereas in the hemisphere where Y < 0, the B_x component is mostly positive. Thus, the direction of the B_x component at lower altitudes is not dependent on the B_x component of the IMFs. This pattern is indicative of draped magnetic field. Although there exists difference between observations and simulation results (especially in Figure 2A), simulation results are generally smooth with distinct features and perturbations in the statistical observational results are inevitable given the low occurrence rate of such events. Note that this is consistent with the hemispheric asymmetry of the B_x component of the draping field line in the lobes of Mars' magnetotail.[40] Moreover, the amplitudes of the magnetic field in the X-Z plane and X-Y plane, as shown in Figure S1, have strong values when in close proximity to Mars, especially on the dayside, indicating the existence of a magnetic barrier/pileup. The magnetic field lines in the hybrid simulation model display an obvious draping pattern (Figures S1E and S1F). Details of the hybrid simulation model can be found in the materials and methods. Joint observations and hybrid simulations suggest that, even with a small cone angle of the IMF, the IMF can still drape around Mars' ionosphere, forming a draped field globally.

Table 1. Rate of same direction between the B_x component in different regions and the B_x component of radial IMFs

Region	$B_{x,\ radial\ IMF} > 0$ & $B_x > 0$ (%)	$B_{x,\ radial\ IMF} < 0$ & $B_x < 0$ (%)
Solar wind	99.93	96.82
Magnetosheath	81.95	81.03
Dayside magnetosphere	60.22	36.29
Nightside magnetosphere	58.84	39.05

Dayside magnetosphere represents regions below MPB and X > 0. Nightside magnetosphere represents regions below MPB and X < 0. The MPB is given by Vignes et al.[39]

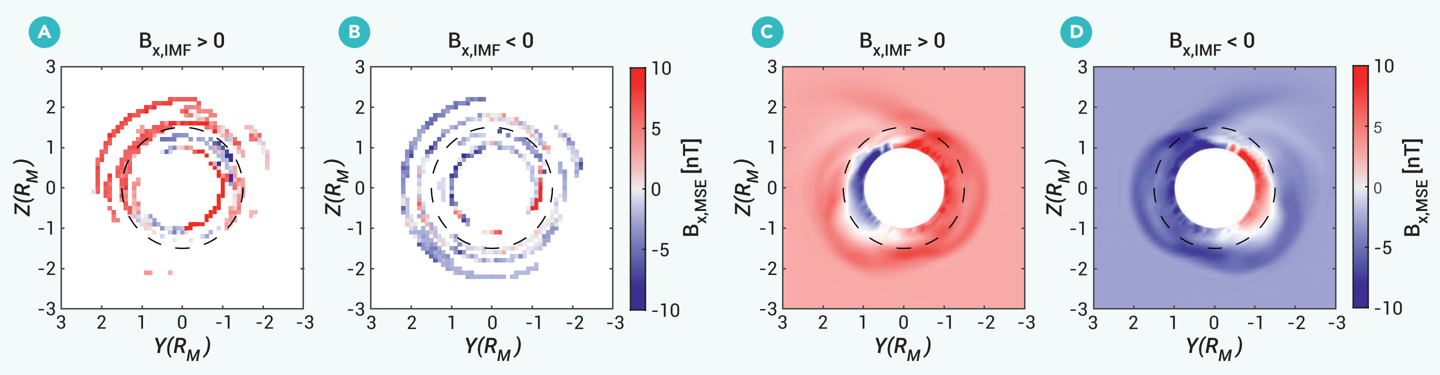

Figure 2. Magnetic field B_x component in MSE coordinate from observations and hybrid simulations Average value of magnetic field B_x component in Y-Z plane in MSE coordinate from observations (A and B) and hybrid simulations (C and D). The dashed circles represent the altitude of 0.5 R_M. In (A and B), the magnetic field B_x component is averaged within $0 \leq X \leq 1.3$ R_M, i.e., within the dayside magnetosphere. (C and D) Magnetic field B_x component in the terminator plane (X = 0). (A and C) Distribution of the magnetic field B_x component when $B_{x,\ \text{radial IMF}} > 0$, and (B and D) that when $B_{x,\ \text{radial IMF}} < 0$. Details of the hybrid simulation model can be found in the materials and methods.

To further investigate details of the draped field in the high-altitude regions and the induced field in the lower-altitude regions, Figure 3 displays a radial IMF event observed simultaneously by the Tianwen-1 and MAVEN satellites at upstream and downstream locations, respectively. Specific satellite trajectories are provided in Figure S2. Given Tianwen-1's trajectory and weak disturbance and low-intensity magnetic field measured by Tianwen-1 (Figure 3A), one can infer that Tianwen-1 was positioned in the solar wind. The cone angle of the IMF mostly remained between −15° and 0, with the B_x component being negative, indicating that the IMF was directed from the Sun toward Mars. Below approximately 500 km (09:23–09:32 UT), MAVEN observed high-density heavy ions, including oxygen ions and molecular ions (Figure 3E), indicating that MAVEN was within Mars' magnetosphere or ionosphere. Near 500 km (approximately 09:32:30 UT), the density of heavy ions rapidly decreased, becoming much lower than that of hydrogen ions, and the thermal pressure of the protons dominated. The proton density in Mars' magnetosheath was higher than that in the magnetosphere,[41] and the magnitude of the magnetic field decreased sharply, while the magnetic field fluctuated more. All these features indicate that the boundary was the MPB. Above the MPB (09:32–09:48 UT), MAVEN observed strong magnetic field disturbances (Figure 3D) and a predominance of hydrogen ions, indicating that MAVEN was in the magnetosheath.

The magnitude of the magnetic field around the MPB was approximately 20 nT, the change in the magnetic field across the MPB was ∼10 nT, and the thickness of the MPB was ∼57 km, which is comparable with the local ion gyroradius (hydrogen ion gyroradius ∼68 km, oxygen ion gyroradius ∼58 km). Thus, the current density around the MPB estimated by Ampere's law is ∼140 nA/m^2 (see materials and methods for a detailed calculation). The plasma pressure gradient across the MPB was ∼0.15 nPa (Figure 3F). The diamagnetic current caused by this plasma pressure gradient around the MPB is estimated as ∼132 nA/m^2 (see the materials and methods for a detailed calculation), which is consistent with that estimated by Ampere's law. This diamagnetic current flows along the upper boundary of the ionosphere from the +E hemisphere to the −E hemisphere, which is consistent with the dayside currents around the MPB in Mars' induced magnetosphere under a nonradial IMF.[42,43] This suggests that the MPB resulted from the diamagnetic current and that the physical nature of the MPB formed in the radial IMF is the same as that formed in the nonradial IMF.

During this whole period, MAVEN remained in the hemisphere where Y > 0 in the MSE coordinates (Figure 3H). If the magnetic field is a draped field, the magnetic field direction should point toward the night with a negative B_x component. Below the MPB, the B_x component of the magnetic field remains negative (Figure 3D) and the magnetic pressure is greater than the thermal pressure (Figure 3F) in higher-altitude regions (09:30:50–09:32:30 UT). This result suggests that the magnetic field in higher-altitude regions was a draped field. The B_x component of the magnetic field changed to positive, which opposed the direction of the IMF in lower-altitude regions (09:23:00–09:30:50 UT), suggesting that the magnetic field in this region was an induced field rather than a draped field. Note that the orbit of MAVEN is not above any strong crustal field regions (Figure S3), indicating that the effect of the crustal field can be ignored in this event.

The statistical results and the case study consistently suggest that the Mars' induced magnetosphere forms in the radial IMF and that the magnetic field consists of a draped field and an induced field. Figure 4 illustrates the whole picture of the draped magnetic field and induced magnetic field in Mars' induced magnetosphere under the radial IMF.

DISCUSSION

The draped field scenario has been well demonstrated by the general draped field pattern model[5,6] with an inverse polarity reversal layer.[4,44] In general, a time-varying external magnetic field induces an electric field in a conductor. In regions with finite conductivity, a current is generated along the direction of the electric field ($\boldsymbol{J} = \sigma \boldsymbol{E}$), where the current density is approximately proportional to both the rate of change of the time-varying magnetic field and the conductivity σ. This analysis primarily accounts for collision effects, i.e., the ohmic term in the generalized Ohm's law. This mechanism corresponds to the dynamo effect in the low-altitude ionosphere (100–200 km). The observed anti-IMF magnetic field at low altitudes should be the result of Mars' ionospheric dynamo when the direction of the IMF changes radially ($\frac{\partial B_x}{\partial t} \neq 0$). The "lifetime" of the induced magnetic field can be interpreted by the magnetic diffusion time $\tau_m \approx \mu_0 \sigma L^2$ derived from the magnetic induction equation, where μ_0 is the permeability of a vacuum and L is the characteristic length scale. Considering that the conductivity $\sigma \approx 10^{-1}$ to 10^0 S/m[45] and $L \approx 100$ km in the low-altitude ionosphere, the magnetic diffuse time is $\sim 10^3$ to 10^4 s. This suggests that the induced magnetic field should diffuse in ∼20 min to 3.5 h, which enables *in situ* observations of the induced magnetic field.

The *in situ* observations and numerical simulations above demonstrated that Mars' induced magnetosphere can still form under the radial IMF, but previous studies have indicated that the induced magnetosphere may degenerate under specific circumstances. Although our simulation model and previous models consider the same physics (both hybrid simulations), there is a significant difference between the models in the resistivity in the ionosphere parameter. The ionospheric resistivity in our model is set to 5×10^3 Ω·m, which is based on measurements of Mars' dayside ionospheric resistivity.[45] When the ionospheric resistivity is much greater (e.g., $\sim 5 \times 10^4$ Ω·m from Zhang et al.[16,46]), the induced magnetosphere can degenerate.[16] Note that we conducted another run in the same hybrid simulation model but with a resistivity of $\sim 5 \times 10^4$ Ω·m, and no draped field or induced field was observed around Mars (not shown here). The importance of Mars' ionosphere as a conductor, which results in deflection of the IMF and the associated draping pattern, has been emphasized by previous studies.[6,37] Generally, a higher ionospheric resistivity suppresses the formation of induced currents, allowing the external magnetic field to penetrate more freely and preventing the development of an effective shielding mechanism. This suggests that the magnitude of ionospheric resistivity is one of the crucial factors that determines whether an induced magnetosphere forms under the radial IMF.

In the flank regions, the bow shock structure can be seen in both observations and simulation results. However, we did not find radial IMF observations near the subsolar point from Tianwen-1 or MAVEN. In the simulations, it can be

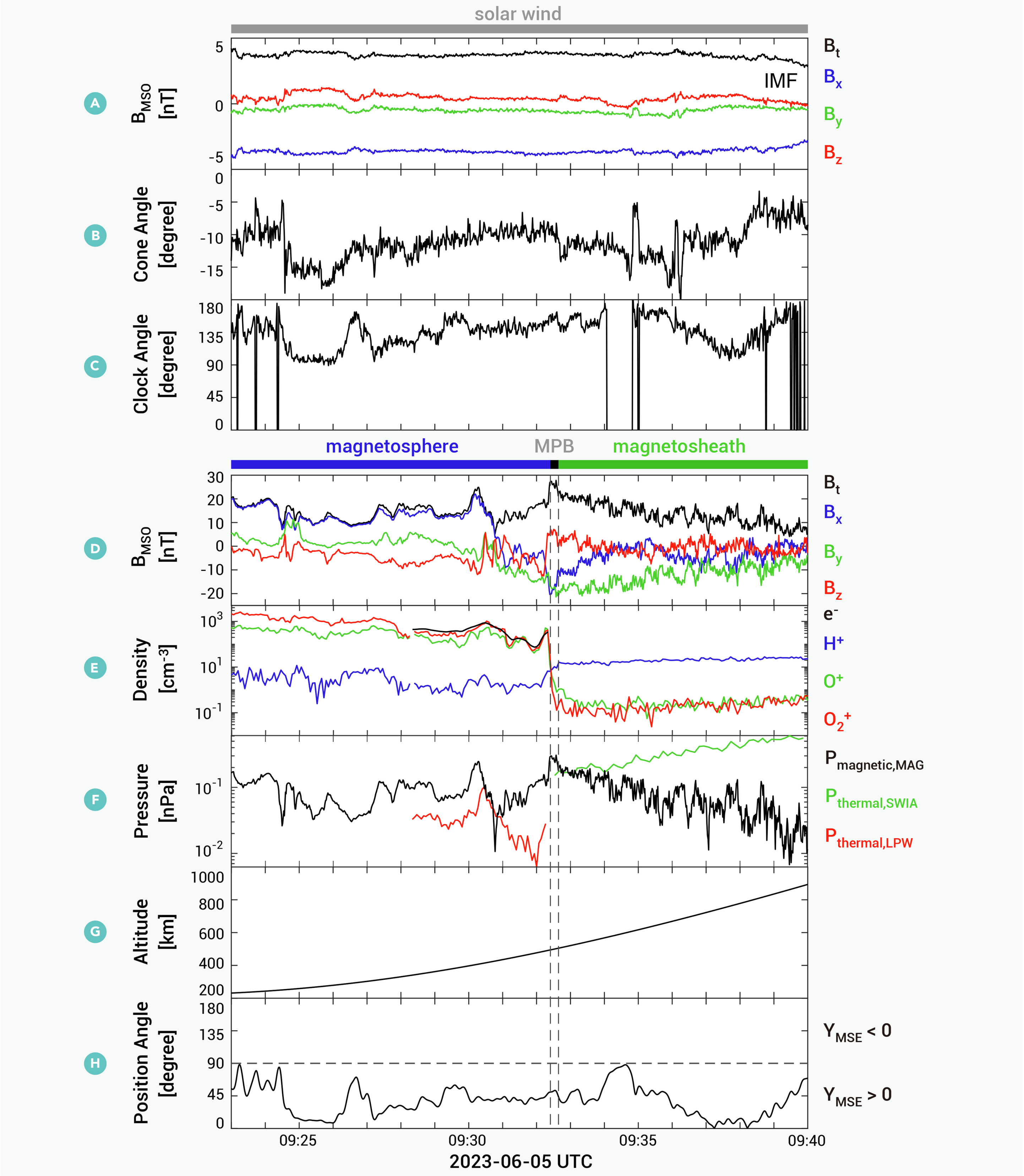

Figure 3. Magnetic field and plasmas measurements by MAVEN, accompanying by the IMF measurements by Tianwen-1 in the time interval of 09:23–09:40 UT on June 5, 2023 (A) IMF in MSO coordinate by Tianwen-1, (B and C) cone angle and clock angle of IMF, (D) magnetic field in MSO coordinate by MAVEN, (E) plasma density, (F) pressure, (G) altitude, and (H) position angle between position vector of MAVEN and IMF projection vector in the Y-Z plane. Position angle is defined as $\cos^{-1}\left(\frac{\boldsymbol{P}_{yz,MAVEN}\cdot\boldsymbol{B}_{yz,Tianwen-1}}{|\boldsymbol{P}_{yz,MAVEN}||\boldsymbol{B}_{yz,Tianwen-1}|}\right)$, where $\boldsymbol{P}_{yz,MAVEN} = [Y, Z]\ R_M$ and $\boldsymbol{B}_{yz,Tianwen-1} = [B_y, B_z]$ nT in MSO coordinates. Note that the angle lower than 90° (higher than 90°) shows that MAVEN is in the hemisphere where Y > 0 (Y < 0) in MSE coordinates.

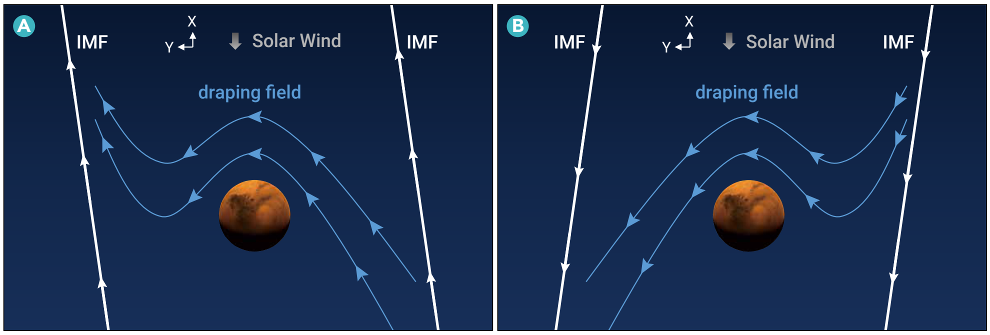

Figure 4. Illustration of the magnetic field in Mars' induced magnetosphere in the case of radial IMFs IMF points (A) toward the Sun, and (B) from the Sun in the X-Y plane in MSE coordinates. The white curves represent the IMF. The blue curves represent draped magnetic fields.

seen that a magnetic cavity structure forms near the subsolar point, and a shock structure still exists upstream of this cavity (Figure S1). Based on the current observations and simulation results, we tend to believe that the shock at the subsolar point exists, because the formation of the draped IMF and induced magnetosphere means that the solar wind carrying the IMF is blocked and deflected. Regarding cross-flow plumes proposed by Zhang et al.,[16] we find several events in the magnetosheath by MAVEN that might be cross-flow plumes, but statistical results and conclusions cannot be drawn at present. In the simulation results, oblique magnetic field structures are visible in the +Z hemisphere in the simulations (Figures 2C and 2D), and these structures are likely indicative of the presence of cross-flow plumes. The formation of cross-flow plumes is reasonable: the radial IMF makes the direction of the convective electric field in the magnetosheath more inclined toward the Y axis and the ion plumes basically align with the convective electric field. Further studies are required to confirm whether these plumes correspond to a strong escape rate.

If the IMF cone angle upper limit was reduced to ±5°, only one event was identified below 1,000 km (Figure S6). This case occurred at altitudes in the range of ~300–500 km on the nightside of Mars (Figures S4G and S5). The IMF magnitude was 2 nT with the field direction pointing away from the Sun ($B_x < 0$; Figure S4C), whereas the magnetic field within the magnetosphere was 5–8 nT with the direction pointing toward the Sun ($B_x > 0$; Figure S4A). The magnitude and direction of the magnetic field suggest that there existed an induced magnetosphere, which is consistent with the results above. Note that the orbit of MAVEN is also not above any strong crustal field regions (Figure S6), indicating that the effect of the crustal field can be ignored in this event.

A radial IMF is a rare event for Mars or other non-magnetized planets in the Solar System. However, for terrestrial exoplanets within the close-in habitable zone of dwarf stars,[47,48] the Parker spiral angle is basically zero and the IMF is generally aligned with the stellar wind. These observations of Mars' induced magnetosphere in the case of radial IMFs provide a rare chance to study stellar wind interactions with these exoplanets.

It should be noted that the number of current observational events is far from sufficient. The occurrence rate of a radial IMF is relatively low at the distance of Mars' orbit around the Sun. On the other hand, the collaborative observation time of the two satellites is relatively short. The limitation of lacking cases of radial IMF leads to discrepancies between observational results and simulation results or theoretical scenarios (such as the region where Y < 0 in Figure 2A). Meanwhile, the observational results in Figure 2 represent the median within the region of $0 < X < 1.3\ R_M$, and the current results cannot fully cover the entire magnetospheric regions (Figures 2A and 2B). Nevertheless, the characteristics of the MPB and draped magnetic field above Mars' ionosphere under a quasi-radial IMF can still be clearly observed. This strongly supports the existence of Mars' induced magnetosphere. When satellites capture more observational events in the future, then it will be possible to further investigate whether the induced magnetosphere under a radial IMF is significantly different from that under other conditions, and explore issues such as changes in ion escape rate from Mars when the IMF becomes radial.

While the regions with strong crustal magnetic fields were excluded to minimize interference, the potential effects of crustal magnetic fields and their interaction with the induced magnetosphere were not fully addressed. Previous studies have demonstrated that the interaction between crustal magnetic fields and the solar wind gives rise to mini-magnetospheres,[49] accompanied by complex global magnetic field topology,[50,51] plasma flow fields,[52] and even magnetospheric dynamic processes analogous to those in Earth's magnetosphere, such as magnetic reconnection.[53,54] Under quasi-radial IMF conditions, the magnetic field of the induced magnetosphere is relatively weak, and the dynamic processes of mini-magnetospheres are expected to exert a stronger influence on the surrounding regions with weak crustal magnetic fields, even dominating the areas originally occupied by the induced magnetosphere.

CONCLUSION

This study clearly reveals the existence of Mars' induced magnetosphere via the joint observations from the Tianwen-1 and MAVEN missions for the first time, which consists of both a draped magnetic field and an induced magnetic field, even in the case of a radial IMF. The IMF drapes around Mars' ionosphere, forming the draped magnetic field driven by the diamagnetic currents in the MPB. This finding indicates that the ion escape rate may not be as high as previously expected for degenerated induced magnetospheres in radial IMFs. Further observations and simulations are needed to explore other physical details of this interaction and to estimate the degree of atmospheric loss due to ion escape.

RESOURCE AVAILABILITY

Materials availability

This study did not generate new unique materials or reagents.

Data and code availability

- The Tianwen-1 data may be applied at CNSA Data Release System (https://moon.bao.ac.cn/mall/MarsDATA) or downloaded from the official website of the MOMAG team (http://space.ustc.edu.cn/dreams/tw1_momag/).
- The MAVEN data used in this study are publicly available at the website: https://lasp.colorado.edu/maven/sdc/public/data/sci/.
- The simulation data used in thi study are available at the website: https://github.com/marc-lin97/Hybrid-simulation-data-of-magnetic-field-Bx-component-around-Mars-under-quasi-radial-IMF.git
- All data are loaded, analyzed, and plotted using MATLAB code package which can be provided for corresponding authors for related further studies.

FUNDING AND ACKNOWLEDGMENTS

We acknowledge the entire Tianwen-1 and MAVEN teams for providing data. This work was supported by the National Natural Science Foundation of China (42574215, 42441811, 42430203, and 42130204). S.H. acknowledges the support from the Opening Project of Joint Laboratory for Planetary Science and Supercomputing (no. CSYYGS-QT-2024-16). We appreciate the useful discussions with Dr. Yue Dong from Wuhan University.

AUTHOR CONTRIBUTIONS

S.H. supervised this work and wrote the manuscript. R.L provided physical ideas, carried out the data analysis, and wrote the manuscript. J.Z. performed the hybrid simulations and wrote the manuscript. Y.W., G.W., and Y.C. confirmed that the released Tianwen-1/MOMAG data are well calibrated. Z.Y., Y.W., K.L., L.C., E.D., M.F., G.W., Y.C., Z.Z., H.W., K.J., and Q.X. revised the manuscript. All authors discussed the results and commented on this manuscript.

DECLARATION OF INTERESTS

The authors declare no competing interests.

SUPPLEMENTAL INFORMATION

It can be found online at https://doi.org/10.1016/j.xinn.2026.101312.

REFERENCES

1. Acuña, M.H., Connerney, J.E.P., Wasilewski, P. et al. (1998). Magnetic field and plasma observations at Mars: Initial results of the Mars global surveyor mission. *Science* **279**:1676–1680. DOI:10.1126/science.279.5357.1676
2. Luhmann, J.G., Ledvina, S.A. and Russell, C.T. (2004). Induced magnetospheres. *Adv. Space Res.* **33**:1905–1912. DOI:10.1016/j.asr.2003.03.031
3. Dubinin, E., Fraenz, M., Woch, J. et al. (2013). Toroidal and poloidal magnetic fields at Venus. Venus Express observations. *Planet. Space Sci.* **87**:19–29. DOI:10.1016/j.pss.2012.12.003
4. Zhang, C., Rong, Z., Klinger, L. et al. (2022). Three-Dimensional Configuration of Induced Magnetic Fields Around Mars. *JGR. Planets* **127**:e2022JE007334. DOI:10.1029/2022JE007334
5. Dubinin, E., Fraenz, M., Modolo, R. et al. (2021). Induced Magnetic Fields and Plasma Motions in the Inner Part of the Martian Magnetosphere. *JGR. Space Physics* **126**:e2021JA029542. DOI:10.1029/2021JA029542
6. Azari, A.R., Abrahams, E., Sapienza, F. et al. (2023). Magnetic Field Draping in Induced Magnetospheres: Evidence From the MAVEN Mission to Mars. *JGR. Space Physics* **128**:e2023JA031546. DOI:10.1029/2023JA031546
7. Wang, M., Guan, Z.J., Xie, L. et al. (2023). Three-dimensional MHD Simulations of the Magnetic Pileup at Mars. *Astron. J.* **166**:179. DOI:10.3847/1538-3881/acf56f
8. Li, S., Lu, H., Cao, J. et al. (2024). Asymmetrical Looping Magnetic Fields and Marsward Flows on the Nightside of Mars. *Geophys. Res. Lett.* **51**:e2024GL109186. DOI:10.1029/2024GL109186
9. Zhang, C., Dong, C., Zhou, H. et al. (2025). Global Energy Transport and Conversion in the Solar Wind-Mars Interaction: MAVEN Observations. *JGR. Planets* **130**:e2025JE009295. DOI:10.1029/2025JE009295
10. Mccomas, D.J., Spence, H.E., Russell, C.T. et al. (1986). The Average Magnetic-Field Draping and Consistent Plasma Properties of the Venus Magnetotail. *J. Geophys. Res.* **91**:7939–7953. DOI:10.1029/JA091iA07p07939
11. Rong, Z.J., Barabash, S., Futaana, Y. et al. (2014). Morphology of magnetic field in near-Venus magnetotail: Venus express observations. *JGR. Space Physics* **119**:8838–8847. DOI:10.1002/2014ja020461
12. Bertucci, C., Hamilton, D.C., Kurth, W.S. et al. (2015). Titan's interaction with the supersonic solar wind. *Geophys. Res. Lett.* **42**:193–200. DOI:10.1002/2014gl062106
13. Cravens, T.E. and Gombosi, T.I. (2004). Cometary magnetospheres: a tutorial. *Adv. Space Res.* **33**:1968–1976. DOI:10.1016/j.asr.2003.07.053
14. Chai, L., Wan, W., Wei, Y. et al. (2019). The Induced Global Looping Magnetic Field on Mars. *Astrophys. J. Lett.* **871**:L27. DOI:10.3847/2041-8213/aaff6e
15. Khurana, K.K., Kivelson, M.G., Stevenson, D.J. et al. (1998). Induced magnetic fields as evidence for subsurface oceans in Europa and Callisto. *Nature* **395**:777–780. DOI:10.1038/27394
16. Zhang, Q., Barabash, S., Holmstrom, M. et al. (2024). Mars's induced magnetosphere can degenerate. *Nature* **634**:45–47. DOI:10.1038/s41586-024-07959-z
17. DeZeeuw, D.L., Nagy, A.F., Gombosi, T.I. et al. (1996). A new axisymmetric MHD model of the interaction of the solar wind with Venus. *J. Geophys. Res.* **101**:4547–4556. DOI:10.1029/95je03363
18. Zhang, T.L., Du, J., Ma, Y.J. et al. (2009). Disappearing induced magnetosphere at Venus: Implications for close-in exoplanets. *Geophys. Res. Lett.* **36**:L20203. DOI:10.1029/2009gl040515
19. Rong, Z.J., Stenberg, G., Wei, Y. et al. (2016). Is the flow-aligned component of IMF really able to impact the magnetic field structure of Venusian magnetotail? *JGR. Space Physics* **121**:10978–10993. DOI:10.1002/2016ja022413
20. Fowler, C.M., Hanley, K.G., McFadden, J. et al. (2022). A MAVEN Case Study of Radial IMF at Mars: Impacts on the Dayside Ionosphere. *JGR. Space Physics* **127**:e2022JA030726. DOI:10.1029/2022JA030726
21. Modolo, R., Chanteur, G.M. and Dubinin, E. (2012). Dynamic Martian magnetosphere: Transient twist induced by a rotation of the IMF. *Geophys. Res. Lett.* **39**:L01106. DOI:10.1029/2011gl049895
22. Romanelli, N., DiBraccio, G., Modolo, R. et al. (2019). Recovery Timescales of the Dayside Martian Magnetosphere to IMF Variability. *Geophys. Res. Lett.* **46**:10977–10986. DOI:10.1029/2019gl084151
23. Wan, W.X., Wang, C., Li, C.L. et al. (2020). China's first mission to Mars. *Nat. Astron.* **4**:721. DOI:10.1038/s41550-020-1148-6
24. Wan, W., Wang, C., Li, C. et al. (2020). The payloads of planetary physics research onboard China's First Mars Mission (Tianwen-1). *Earth Planet. Phys.* **4**:331–332. DOI:10.26464/epp2020052
25. Wang, Y., Zhang, T., Wang, G. et al. (2023). The Mars orbiter magnetometer of Tianwen-1: in-flight performance and first science results. *Earth Planet. Phys.* **7**:216–228. DOI:10.26464/epp2023028
26. Jakosky, B.M., Lin, R.P., Grebowsky, J.M. et al. (2015). The Mars Atmosphere and Volatile Evolution (MAVEN) Mission. *Space Sci. Rev.* **195**:3–48. DOI:10.1007/s11214-015-0139-x
27. Cheng, L., Lillis, R., Wang, Y. et al. (2023). Martian Bow Shock Oscillations Driven by Solar Wind Variations: Simultaneous Observations From Tianwen-1 and MAVEN. *Geophys. Res. Lett.* **50**:e2023GL104769. DOI:10.1029/2023GL104769
28. Liu, K., Hao, X., Li, Y. et al. (2020). Mars Orbiter magnetometer of China's First Mars Mission Tianwen-1. *Earth Planet. Phys.* **4**:384–389. DOI:10.26464/epp2020058
29. Connerney, J.E.P., Espley, J., Lawton, P. et al. (2015). The MAVEN Magnetic Field Investigation. *Space Sci. Rev.* **195**:257–291. DOI:10.1007/s11214-015-0169-4
30. Mitchell, D.L., Mazelle, C., Sauvaud, J.A. et al. (2016). The MAVEN Solar Wind Electron Analyzer. *Space Sci. Rev.* **200**:495–528. DOI:10.1007/s11214-015-0232-1
31. McFadden, J.P., Kortmann, O., Curtis, D. et al. (2015). MAVEN SupraThermal and Thermal Ion Compostion (STATIC) Instrument. *Space Sci. Rev.* **195**:199–256. DOI:10.1007/s11214-015-0175-6
32. Zou, Z., Wang, Y., Zhang, T. et al. (2023). In-flight calibration of the magnetometer on the Mars orbiter of Tianwen-1. *Sci. China Technol. Sci.* **66**:2396–2405. DOI:10.1007/s11431-023-2401-2
33. Wang, G., Xiao, S., Wu, M. et al. (2024). Calibration of the Zero Offset of the Fluxgate Magnetometer on Board the Tianwen-1 Orbiter in the Martian Magnetosheath. *JGR. Space Physics* **129**:e2023JA031757. DOI:10.1029/2023JA031757
34. Holmberg, M.K.G., André, N., Garnier, P. et al. (2019). MAVEN and MEX Multi-instrument Study of the Dayside of the Martian Induced Magnetospheric Structure Revealed by Pressure Analyses. *J Geophys Res-Space* **124**:8564–8589. DOI:10.1029/2019ja026954
35. Morschhauser, A., Lesur, V. and Grott, M. (2014). A spherical harmonic model of the lithospheric magnetic field of Mars. *JGR. Planets* **119**:1162–1188. DOI:10.1002/2013je004555
36. Jarvinen, R., Brain, D.A., Modolo, R. et al. (2018). Oxygen Ion Energization at Mars: Comparison of MAVEN and Mars Express Observations to Global Hybrid Simulation. *JGR. Space Physics* **123**:1678–1689. DOI:10.1002/2017ja024884
37. Zhou, J., Liu, K., Jarvinen, R. et al. (2024). Hybrid Simulations of the Martian Magnetotail Twist. *Astrophys. J.* **976**:7. DOI:10.3847/1538-4357/ad8159
38. Sahraoui, F., Belmont, G. and Rezeau, L. (2003). Hamiltonian canonical formulation of Hall-magnetohydrodynamics: Toward an application to weak turbulence theory. *Phys. Plasmas* **10**:1325–1337. DOI:10.1063/1.1564086
39. Vignes, D., Mazelle, C., Rme, H. et al. (2000). The solar wind interaction with Mars:: locations and shapes of the bow shock and the magnetic pile-up boundary from the observations of the MAG/ER experiment onboard Mars Global Surveyor. *Geophys. Res. Lett* **27**:49–52. DOI:10.1029/1999gl010703
40. Wen, Y., Rong, Z., Nilsson, H. et al. (2025). Statistical Investigations of the Radial Interplanetary Magnetic Field Component Impact on the Magnetotail Current Sheet Structure of Mars: MAVEN Observations. *Astrophys. J.* **993**:201. DOI:10.3847/1538-4357/ae11b3
41. Wang, J., Lee, L.C., Xu, X. et al. (2020). Plasma and magnetic-field structures near the Martian induced magnetosphere boundary: I. Plasma depletion region and tangential discontinuity. *Astron Astrophys* **642**. DOI:10.1051/0004-6361/201936201
42. Ramstad, R., Brain, D.A., Dong, Y. et al. (2020). The global current systems of the Martian induced magnetosphere. *Nat. Astron.* **4**:979–985. DOI:10.1038/s41550-020-1099-y
43. Gao, J., Li, S., Mittelholz, A. et al. (2024). Two distinct current systems in the ionosphere of Mars. *Nat. Commun.* **15**:9704. DOI:10.1038/s41467-024-54073-9
44. Romanelli, N., Bertucci, C., Gómez, D. et al. (2015). Dependence of the location of the Martian magnetic lobes on the interplanetary magnetic field direction: Observations from Mars Global Surveyor. *J Geophys Res-Space* **120**:7737–7747. DOI:10.1002/2015ja021359
45. Opgenoorth, H.J., Dhillon, R.S., Rosenqvist, L. et al. (2010). Day-side ionospheric conductivities at Mars. *Planet. Space Sci.* **58**:1139–1151. DOI:10.1016/j.pss.2010.04.004
46. Zhang, Q., Holmström, M. and Wang, X.D. (2023). Effects of ion composition on escape and morphology on Mars. *Ann Geophys-Germany* **41**:375–388. DOI:10.5194/angeo-41-375-2023
47. Khodachenko, M.L., Ribas, I., Lammer, H. et al. (2007). Coronal Mass Ejection (CME) activity of low mass M stars as an important factor for the habitability of terrestrial exoplanets. I. CME impact on expected magnetospheres of earth-like exoplanets in close-in habitable zones. *Astrobiology* **7**:167–184. DOI:10.1089/ast.2006.0127
48. Lammer, H. (2007). M star planet habitability - Preface. *Astrobiology* **7**:27–29. DOI:10.1089/ast.2006.0123
49. Fan, K., Wei, Y., Fraenz, M. et al. (2023). Observations of a Mini-Magnetosphere Above the Martian Crustal Magnetic Fields. *Geophys. Res. Lett.* **50**:e2023GL103999. DOI:10.1029/2023GL103999
50. Xu, S., Weber, T., Mitchell, D.L. et al. (2019). A Technique to Infer Magnetic Topology at Mars and Its Application to the Terminator Region. *JGR. Space Physics* **124**:1823–1842. DOI:10.1029/2018ja026366
51. Weber, T., Brain, D., Xu, S. et al. (2020). The Influence of Interplanetary Magnetic Field Direction on Martian Crustal Magnetic Field Topology. *Geophys. Res. Lett.* **47**:e2020GL087757. DOI:10.1029/2020GL087757
52. Fan, K., Fraenz, M., Wei, Y. et al. (2020). Deflection of Global Ion Flow by the Martian Crustal Magnetic Fields. *Astrophys. J. Lett.* **898**:L54. DOI:10.3847/2041-8213/aba519
53. Harada, Y., Halekas, J.S., DiBraccio, G.A. et al. (2018). Magnetic Reconnection on Dayside Crustal Magnetic Fields at Mars: MAVEN Observations. *Geophys. Res. Lett.* **45**:4550–4558. DOI:10.1002/2018gl077281
54. Lin, R.T., Huang, S.Y., Yuan, Z.G. et al. (2024). Direct Observations of Magnetic Reconnections at the Magnetopause of the Martian Mini-Magnetosphere. *Geophys. Res. Lett.* **51**:e2024GL108880. DOI:10.1029/2024GL108880

The Innovation, Volume 7

Supplemental Information

Mars' induced magnetosphere can form under radial interplanetary magnetic field

Rentong Lin, Shiyong Huang, Jingyi Zhou, Yuming Wang, Zhigang Yuan, Eduard Dubinin, Markus Fränz, Haoyu Lu, Kaijun Liu, Lihui Chai, Yihui Song, Guoqiang Wang, Yutian Chi, Honghong Wu, Kui Jiang, Qiyang Xiong, and Zhuxuan Zou

Supplementary Information

Supplemental Figures

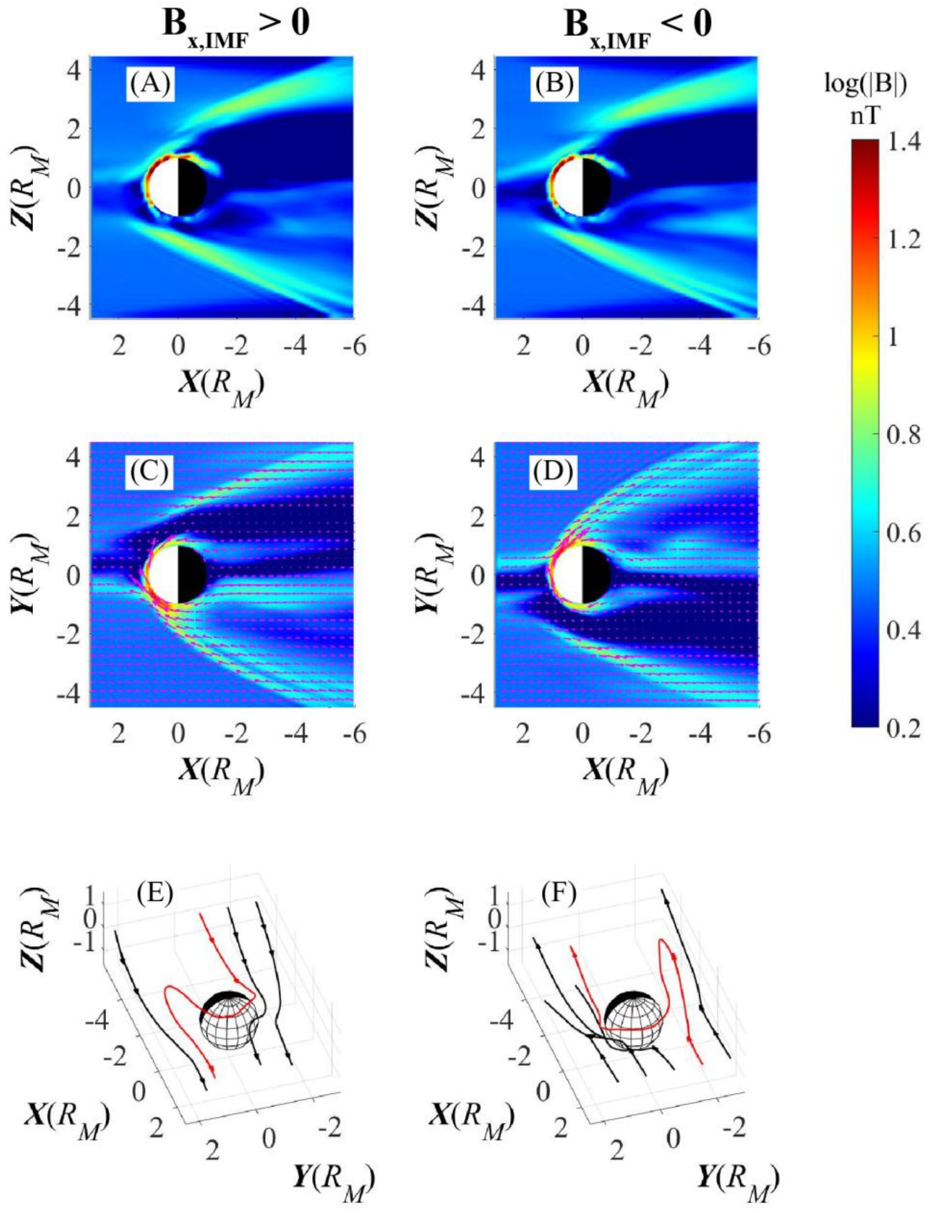

Figure S1. Magnitude of magnetic field in X-Z plane and X-Y plane in MSE coordinate and magnetic field lines from hybrid simulations. Panel a presents distribution of magnetic field magnitude when $B_{x,IMF} > 0$ and Panel b presents that when $B_{x,IMF} < 0$ in X-Z plane (Y = 0). Panel c presents distribution of magnetic field magnitude when $B_{x,IMF} > 0$ and Panel d presents that when $B_{x,IMF} < 0$ in X-Y plane (Z = 0). Panel e presents distribution of magnetic field lines when $B_{x,IMF} > 0$. The magnetic field lines originate from a group of points with the X = -6 R_M and Z = 0 R_M but different Y values. And Panel f presents distribution of magnetic field lines when $B_{x,IMF} < 0$. The magnetic field lines originate from a group of points with the X = 3 R_M and Z = 0 R_M but different

Y values. The red streamlines in panel e and f represent draped magnetic fields, corresponding to the blue curves in Fig 5.

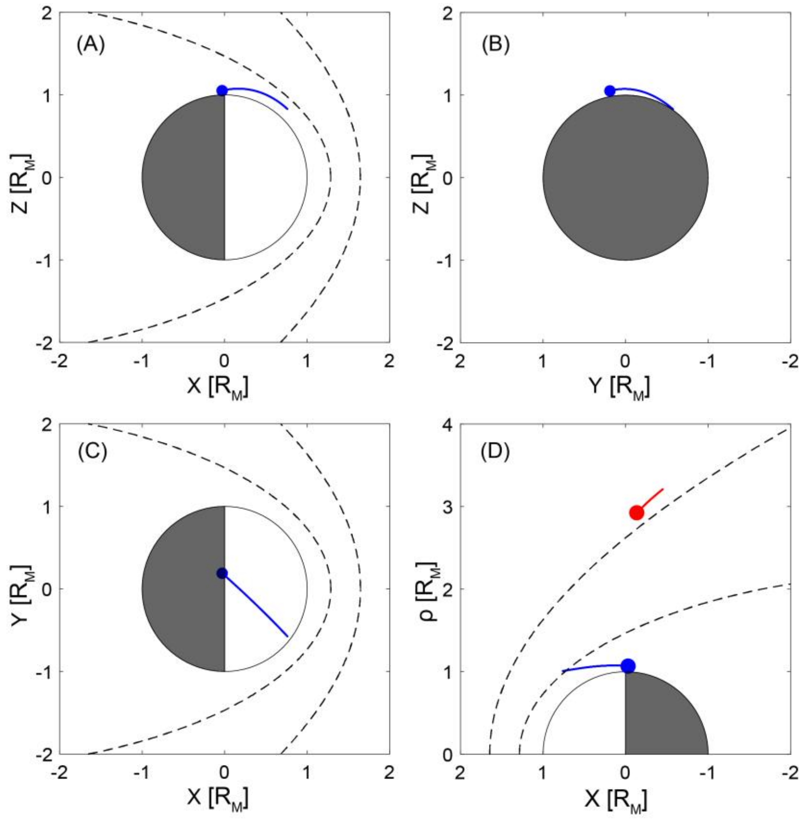

Figure S2. Orbits of Tianwen-1 and MAVEN orbiters in the time interval of 09:23-09:40 UT on June 5, 2023. Orbits of MAVEN (a) in X-Z plane from the dawn, (b) in Y-Z plane viewed from the nightside, (c) in X-Y plane from the north pole, and (d) in X-ρ plane, where $\rho = \sqrt{y^2 + z^2}$. The blue curve represents orbit of MAVEN and the red one represents orbit of Tianwen-1. The blue and red dots represent start of the orbit of orbiters, respectively. The two dashed curves represent the bow shock and the induced magnetosphere boundary from the model (Vignes et al., 2000), respectively. The circle in (a-c) and the half of black circle in (d) represents Mars.

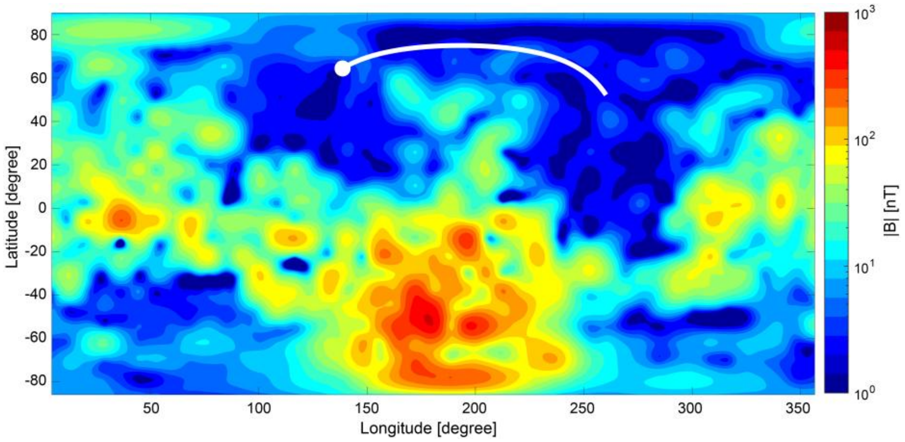

Figure S3. The contour map represents the magnitude of crustal magnetic field at altitude of 185 km by model (Morschhauser et al., 2014). The white dot represents the start of the orbit of MAVEN at 09:15 UT and the white curve represents the orbit of MAVEN in the time interval of 09:15-09:48 UT on June 5, 2023.

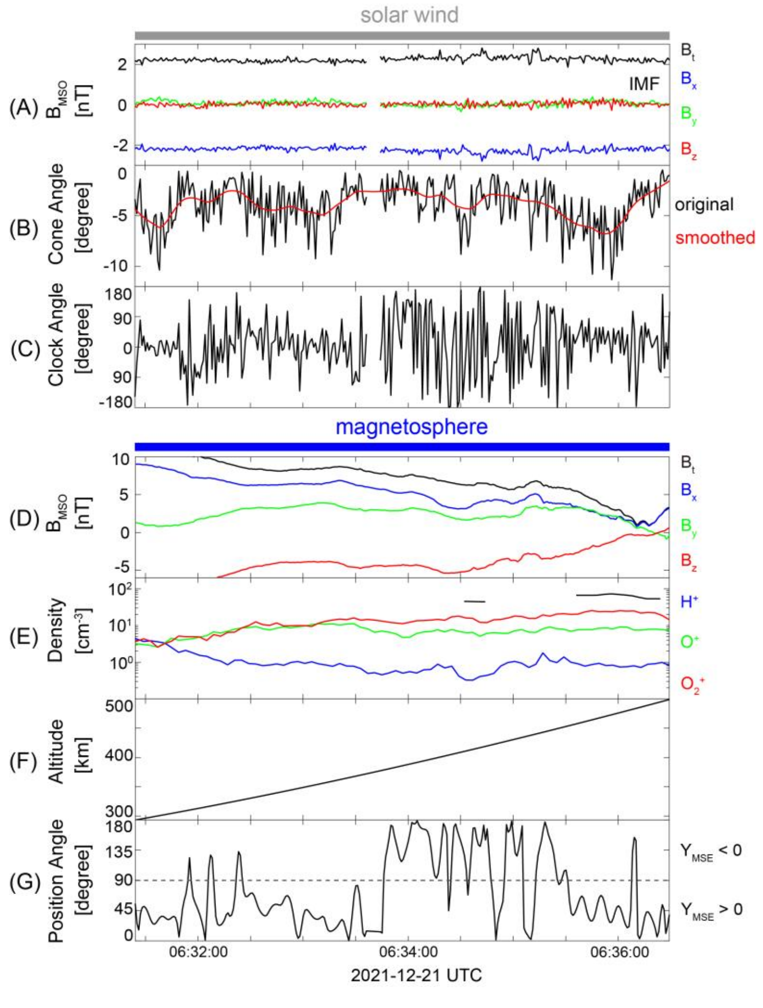

Figure S4. Magnetic field and plasmas measurements by MAVEN, accompanying by

the interplanetary magnetic field (IMF) measurements by Tianwen-1 in the time interval of 06:31:25-06:36:30 UT on December 21, 2021. (a) IMF in MSO coordinate by Tianwen-1, (b-c) cone angle and clock angle of IMF, (d) magnetic field in MSO coordinate by MAVEN, (e) ion density, (f) altitude, and (g) position angle between position vector of MAVEN and IMF projection vector in the Y-Z plane. The red curve in panel (b) represents the cone angle of IMF smoothed over 30 data points with 1Hz sampling rate of magnetic field. Position angle is defined as $\cos^{-1}\left(\frac{\boldsymbol{P}_{yz,MAVEN}\cdot\boldsymbol{B}_{yz,Tianwen-1}}{|\boldsymbol{P}_{yz,MAVEN}||\boldsymbol{B}_{yz,Tianwen-1}|}\right)$, where $\boldsymbol{P}_{\mathrm{yz,MAVEN}} = [\mathrm{Y, Z}]\ \mathrm{R_M}$ and $\boldsymbol{B}_{\mathrm{yz,Tianwen\text{-}1}} = [B_{\mathrm{y}}, B_{\mathrm{z}}]$ nT in MSO coordinate. Note that the angle lower than 90 degrees (higher than 90 degrees) represents that MAVEN is in hemisphere where Y > 0 (Y < 0) in MSE coordinate.

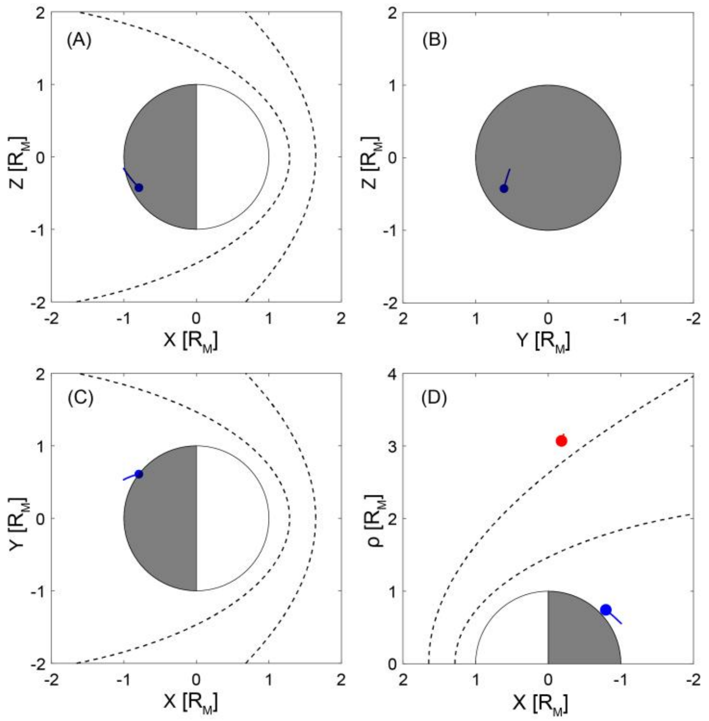

Figure S5. Orbits of Tianwen-1 and MAVEN orbiters in the time interval of 06:31:25-06:36:30 UT on December 21, 2021. Orbits of MAVEN (a) in X-Z plane from the dawn, (b) in Y-Z plane viewed from the nightside, (c) in X-Y plane from the north pole, and (d) in X-ρ plane, where $\rho = \sqrt{y^2 + z^2}$. The blue curve represents orbit of MAVEN and the red one represents orbit of Tianwen-1. The blue and red dots represent start of the orbit of orbiters, respectively. The two dashed curves represent the bow shock and the induced magnetosphere boundary from the model (Vignes et al., 2000), respectively. The circle in (a-c) and the half of black circle in (d) represents Mars.

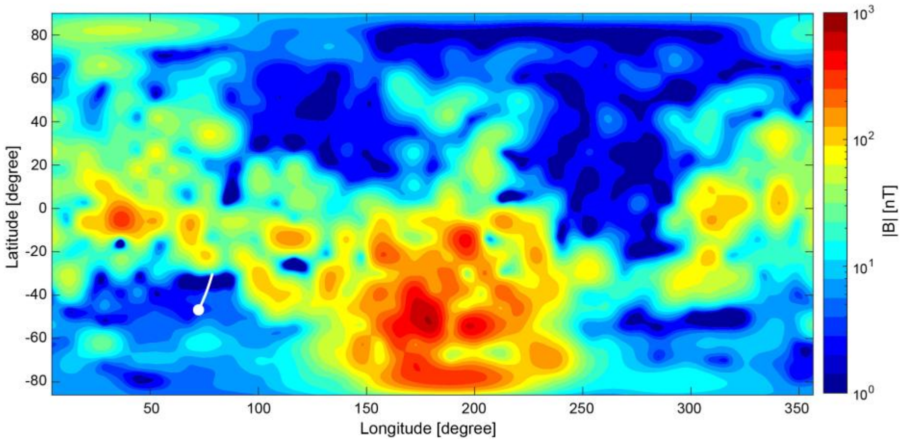

Figure S6. The contour map represents the magnitude of crustal magnetic field at altitude of 185 km by model (Morschhauser et al., 2014). The white dot represents the start of the orbit of MAVEN at 06:31:25 UT and the white curve represents the orbit of MAVEN in the time interval of 06:31:25-06:36:30 UT on December 21, 2021.
